# Approaching Sixfold Chemical Bonding: Metal Dimers MM' Involving Group 4–8 Transition Metals and the Actinides Th–Np. Additive Covalent Radii $r_{6}$

**DOI:** 10.1002/chem.70818

**Published:** 2026-02-25

**Authors:** Pekka Pyykkö, Michiko Atsumi

**Affiliations:** ^1^ Department of Chemistry Faculty of Science University of Helsinki Helsinki Finland; ^2^ Hylleraas Centre for Quantum Molecular Sciences Department of Chemistry University of Oslo Oslo Norway

**Keywords:** Actinides, covalent radii, multiple bonds, Transition metals

## Abstract

A set of additive covalent radii is fitted to a combination of experimental data, accurate ab initio computations, and more approximate DFT MM' bond lengths, R, with 12 valence electrons in approximately ${\left(1\sigma \right)}^{2}{\left(2\sigma \right)}^{2}\pi^{4}\delta^{4}$ molecular orbitals. These $r_{6}$ are typically 10–20 pm shorter than the previous triple‐bond radii $r_{3}$. Values for the elements Ti–Fe, Zr–Ru, Hf–Os, and Th–Np are fitted. Extrapolated values for suitable cases in Group 3 (including rare earths) and Group 9 plus Pu are also proposed.

## Graphical abstract

Twelve‐valence‐electron transition metal dimers MM', like diatomic Mo_2_, can have nearly sixfold bonds. Values of $r_{6}$ for over 20 elements from Sc to Pu are proposed.

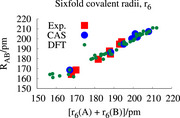

## 1 Introduction

Atoms are not hard spheres. Yet, chemical bond lengths, R, can often be approximated as

(1) $$R_{AB}=r_{A}+r_{B}$$

for covalent bonds of a given type. We have recently proposed such new radius sets $r_{1}$ [1], $r_{2}$ [2], and $r_{3}$ [3] for single, double, and triple bonds, or for tetrahedrally bound crystals $r_{T}$ [4], respectively, with a review and summary [5].

In certain cases one can go further. As quoted by Roos et al. [6], Cotton and coworkers [7] identified the quadruple Cr‐Cr bond in the [Re_2_Cl_8_]^2−^ ion. See also [8]. Additionally, the linear, open‐shell FUUF is assigned as an approximate quadruple bond [9], although its U‐U of 242.8 pm is above 2· $r_{3}$ = 236 pm. Further examples of quadruple and quintuple M‐M bonds exist in organometallic chemistry.

One can approach a formally sixfold MM' bond in the twelve‐valence‐electron (12‐VE) transition‐metal dimers MM', such as the experimentally known Mo_2_ [10].

In a single‐configuration picture they could have a

(2) $${\left(1\sigma \right)}^{2}{\left(2\sigma \right)}^{2}{\left(1\pi \right)}^{4}{\left(1\delta \right)}^{4},X^{1}\Sigma .$$

electron configuration. With an inner and outer σ bond, such a system could approach a sixfold (*s*extuple, hextuple) bond. An instant reservation in any multiconfiguration description, such as CASPT2, is that the occupation of the antibonding partners may diminish the actual effective bond order, EBO. As an example, Mn–U in a polynuclear complex would be estimated full fivefold bond though EBO was only 4.03. [11] For a review of the early experimental literature of these 12‐VE MM' species, see Krechkivska and Morse [12]. An earlier, authoritative review on clusters of transition‐metal atoms, notably Cr_2_ and Mo_2_, was given by Morse [13].

Experimental work on such diatomics was notably done in the groups of Efremov, Bondybey, and Morse. A well‐known case was the Cr_2_ dimer [14, 15], where the R may still approach that of a high bond‐order, but the dissociation energy $D_{e}$ is small. For an early discussion of the homonuclear dimers, see Roos et al. [6]. For both Mo_2_ and W_2_ their effective bond order was 5.2. For Cr_2_ [16] their EBO is only 4.51. Wang et al. [17] considered the Group‐6 M_2_ dimers and found the M = Cr‐W cases to be close to sixfold, but the M = Sg case only fourfold, due to the relativistic stabilization of the 7s shell.

Ruipérez et al. [18] found in MoU an EBO of 5.5 and for CrU and WU 5.3.

## 2 Methods

We shall follow our previous “broad‐brush” method of mixing available accurate experimental data, accurate ab initio data, and more approximate scalar relativistic DFT results. The Gaussian 16 [19] and ORCA [20]software were used in new calculations.

The previous least‐square fitting program was updated. The iterations were started from Group‐6 MM' dimers and were then extended to other (n−1)d metals and also the actinides Th–Np in 12‐VE diatomics.

Concerning the three species ${\mathrm{VCr}}^{-}$, ${\mathrm{VMo}}^{-}$. and ${\mathrm{VW}}^{-}$, we have kept our own DFT R where the electron configuration (2) was imposed. We have omitted the CASPT2 results of Ruipérez et al. [21], where the result was actually found to be 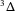, 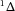, and 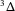, respectively.

## 3 Results

**FIGURE 1:**
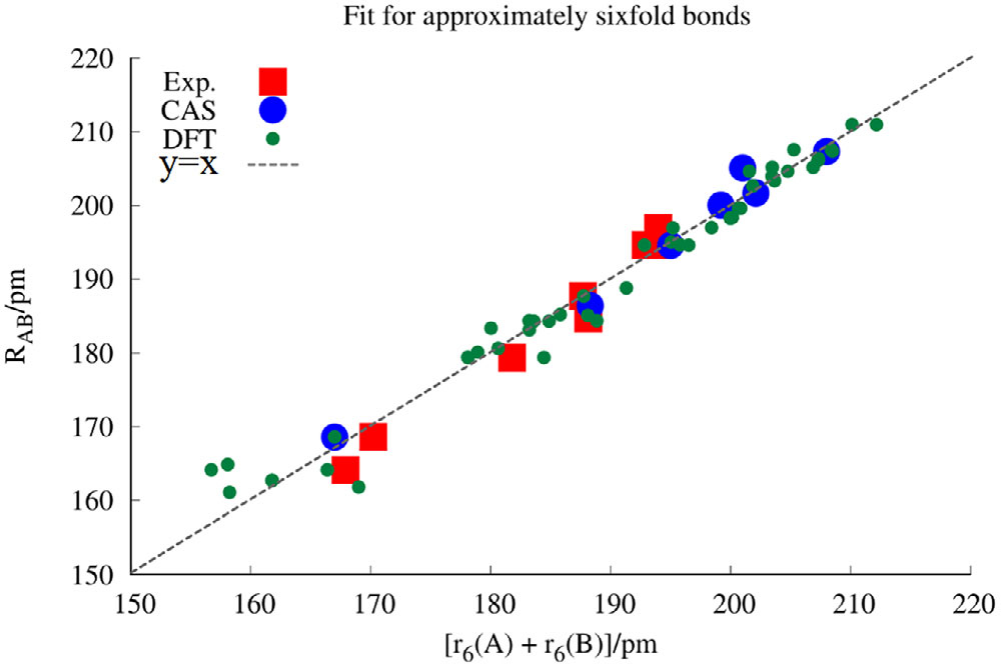
The $R_{AB}$ as function of $r_{A}$ + $r_{B}$.

The input data set is shown in Table 1. The original fit to these data is shown in Figure 1.

**TABLE 1:** Input distances, R (in pm).

Z1	Z2	Species	R(pm)	Source	Z1	Z2	Species	R(pm)	Source
24	24	Cr_2_	156.72	pw^a^	22	26	TiFe	170.24(3)	Exp $R_{0}$ [22]
24	24	Cr_2_	167.88	Exp $R_{e}$ [15]	23	26	${\mathrm{VFe}}^{+}$	161.78	pw^a^
24	24	Cr_2_	166	CASPT2 [6]	24	43	${\mathrm{CrTc}}^{+}$	178.1	pw^a^
42	42	Mo_2_	192.9	Exp [23]	25	91	MnPa	184.87	pw^a^
42	42	Mo_2_	194.0(9)	Exp [24]	25	92	${\mathrm{MnU}}^{+}$	180.04	pw^a^
42	42	Mo_2_	195	Calc [10]	26	40	FeZr	187.75	pw^a^
42	42	Mo_2_	196.51	pw^a^	26	40	FeZr	187.685(20)	Exp $R_{0}$ [12]
74	74	W_2_	201	CASPT2 [25]	26	90	FeTh	195.05	pw^a^
74	74	W_2_	204.77	pw^a^	41	44	${\mathrm{NbRu}}^{+}$	199.99	pw^a^
74	74	W_2_	201.56	pw^b^	42	75	${\mathrm{MoRe}}^{+}$	200.14	pw^a^
24	42	CrMo	181.82(15)	Exp $R_{e}$ [22, 26]	73	76	${\mathrm{TaOs}}^{+}$	207.31	pw^a^
24	42	CrMo	178.11	pw^a^	26	72	FeHf	191.32	pw^a^
24	74	CrW	188.14(4)	Exp $R_{0}$ [27]	44	72	RuHf	208.4	pw^a^
24	74	CrW	183.21	pw^a^	72	76	HfOs	212.16	pw^a^
42	74	MoW	200.84	pw^a^	24	42	CrMo	184.45	pw^b^
42	74	MoW	199.2	CASPT2 [21]	24	74	CrW	166.39	pw^b^
24	92	CrU	188.3	CASPT2 [18]	41	42	${\mathrm{NbMo}}^{-}$	195.2	pw^b^
42	92	MoU	202.1	CASPT2 [18]	42	74	MoW	200.65	pw^b^
74	92	WU	208	CASPT2 [18]	44	74	RuHf	205.28	pw^b^
23	24	${\mathrm{VCr}}^{-}$	158.09	pw^a^	72	76	HfOs	210.13	pw^b^
23	24	${\mathrm{VCr}}^{-}$	174.5	CASPT2 [21]^c^	73	75	TaRe	203.48	pw^b^
23	42	${\mathrm{VMo}}^{-}$	178.93	pw^a^	22	26	TiFe	167	DFT [28]
23	74	${\mathrm{VW}}^{-}$	188.1	pw^a^	23	43	VTc	176.3	pw^b^
23	25	VMn	158.1	pw^a^					
23	26	${\mathrm{VFe}}^{+}$	161.78	pw^a^					
24	25	${\mathrm{CrMn}}^{+}$	158.24	pw^a^					
24	75	${\mathrm{CrRe}}^{+}$	183.22	pw^a^					
24	93	${\mathrm{CrNp}}^{+}$	180.63	pw^a^					
41	42	${\mathrm{NbMo}}^{-}$	194.0(25)	Exp [29]					
41	43	NbTc	198.42	pw^a^					
41	44	${\mathrm{NbRu}}^{+}$	199.99	pw^a^					
42	43	${\mathrm{MoTc}}^{+}$	195.8	pw^a^					
73	75	TaRe	206.88	pw^a^					
73	76	${\mathrm{TaOs}}^{+}$	207.31	pw^a^					
74	75	${\mathrm{WRe}}^{+}$	203.67	pw^a^					

*Note*: A 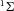 ground state was imposed in the present calculations.

Abbreviation: pw, present work.

^a^ Gaussian 16, B3LYP.

^b^ ORCA BHandHLYP.

**FIGURE 2:**
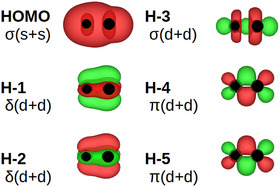
An example of the molecular orbitals (Table 1): VTc.

An example of the MO:s is given in Figure 2. Note the two σ orbitals, HOMO and H‐3, the δ orbitals H‐1,2, and the π orbitals H‐4,5.

**FIGURE 3:**
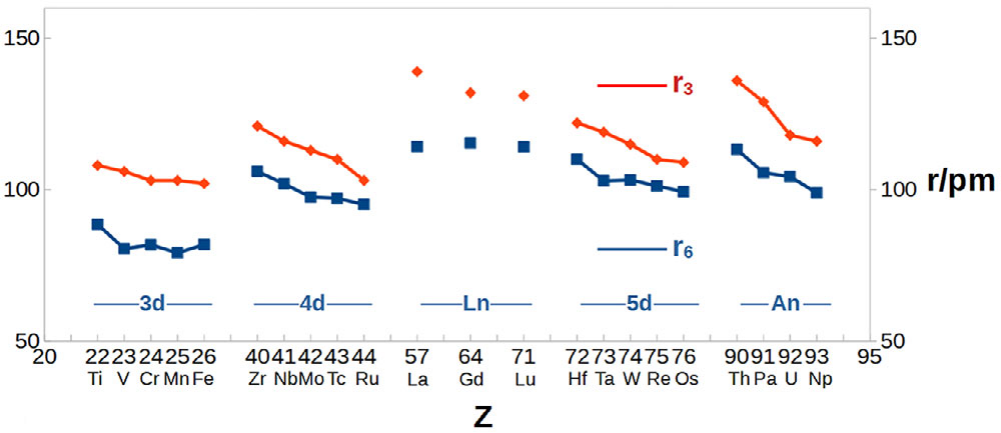
A comparison of previous $r_{3}$ and present $r_{6}$.

The obtained results from the self‐consistent fit are given in Table 2. As seen from Figure 3, the present $r_{6}$ are typically 10–20 pm shorter than the corresponding $r_{3}$. The mean‐square deviation of the fit was 2.4 pm.

**TABLE 2:** $r_{6}$‐Values from a self‐consistent fit.

Group 4	Group 5	Group 6	Group 7	Group 8
Ti 88.6	V 82.8	Cr 82.1	Mn 79.2	Fe 80.1
(108)	(106)	(103)	(103)	(102)
Zr 107.7	Nb 99.5	Mo 97.4	Tc 97.5	Ru 99.0
(121)	(116)	(113)	(110)	(103)
Hf 109.0	Ta 104.6	W 102.3	Re 101.6	Os 102.8
(122)	(119)	(115)	(110)	(109)
Th 115.0	Pa 105.4	U 104.4	Np 98.5	
(136)	(129)	(118)	(116)	

*Note*: In the parentheses, $r_{3}$ values are shown.

They further allowed their extrapolation to the neighboring elements in Table 3. Concerning the rare earths Sc, Y, La, and Lu, the condition (2) was imposed, in agreement with experiment for ScCo and CoY. For GdIr, the same MOs were filled, with an additional half‐filled Gd $4f^{7}$ shell [30].

**TABLE 3:** Input distances, R, and resulting $r_{6}$ (in pm) for further cases.

Z1	Z2	Species	R	Source	$r_{6}$(**M**)
22	27	Ti**Co** 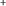	172.56	pw^a^	**84.0**
22	45	Ti**Rh** 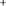	189.34	pw^a^	**100.7**
22	77	Ti**Ir** 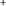	193.41	pw^a^	**104.8**
21	27	**ScCo**	181.21(10)	Exp. $R_{0}$ [31]	97.2
21	27	**ScCo**	180.05	pw^a^	95.85
		**Sc(av)**			**96.1**
22	94	TiPu	199.41	pw^a^	110.8
23	94	${\mathrm{VPu}}^{+}$	187.56	pw^a^	104.8
41	94	${\mathrm{NbPu}}^{+}$	210.06	pw^a^	110.6
		**Pu**(av.)			**108.7**
27	39	Co**Y**	201.61	pw^a^	117.6
27	39	Co**Y**	198.30(8)	Exp. $R_{0}$ [31]	114.3
		**Y(av.)**			**116.0**
27	57	**La**Co	198.45	pw^a^	**114.5**
27	71	**Lu**Co	198.36	pw^a^	**114.4**
39	77	YIr	222.72	pw^a^	
39	77	YIr	221	pw $\Sigma r_{6}$	
64	77	**Gd** ${\mathrm{Ir}}^{+}$	221.44	pw^a^, ^c^	116.4
64	77	**Gd** ${\mathrm{Ir}}^{+}$	219.64	pw^b^, ^c^	114.6
		**Gd**(av.)			**115.5**

^a^ Gaussian 16, B3LYP.

^b^ ORCA BHandHLYP.

^c^ S=7/2 imposed.

## 4 Discussion

### 4.1 Comparison With the Morse Multiple‐Bonding Radii

The group of M. D. Morse have derived a set of additive “multiple bond radii,” based on experimental R values and having an accuracy of 1.2 pm [32]. They are using a reliable data base but a mixture of electron configurations while we are using a mixed data base for systems that more or less satisfy Equation (2). For Mo and W, the two approaches could agree, and indeed do so, as seen in Table 4. The deviations between the two approaches show up for Cr in the South‐Western corner of Figure 1.

**TABLE 4:** Comparison of present $r_{6}$ (in pm) and the Morse $r_{M}$.

M	$r_{6}$	Method	$r_{M}$	Ref.
V	82.8	Exp.	89.19	[32]^a^
Cr	82.9	Exp.	84.40	[32]
Nb	99.5	Exp.	104.24	[32]^a^
Mo	97.4	Exp.	97.25	[32]
W	102.3	Exp.	103.7	[27]

^a^ Heteronuclear, open‐shell 11‐VE case.

The Nagarajan and Morse [31] values for V and Nb are longer than ours because they considered 11‐VE neutrals and not 12‐VE ions. Their experimental r(Cr) value is more “correct” than ours because we included single‐configuration DFT calculations, thus imposing Equation (2).

We also note that for the 10‐VE metastable species $U_{2}^{2+}$, an inner CASPT2 minimum occurs at about R = 230 pm, while $2r_{3}=236$ and $2r_{6}=209$ pm [33].

### 4.2 Further Comments

Further comparisons of Group 5–7 and 6–6 distances in both diatomic 12‐VE systems and larger organometallics were published by Dong et al. [34].

As a further example on the forced shortness of a Cr_2_, with imposed MO structure (2), we quote our fully numerical Hartree–Fock–Slater R of 156 pm [35].

The present $r_{1}$–$r_{3}$ radii have been used as training data by Mahdavi et al. [36].

The Cr_2_ – W_2_ have been treated by Natural Orbital Functional Theory (NOFT) by Ruipérez et al. [37].

We note that the calculated R(TM‐An) in the (TM)(An)L complexes of Hu et al. [11], TM = Cr–Fe; An = U–Pu, are only slightly (<10 pm) above our $\Sigma r_{6}$, and thus approach sixfold bonding.

### 4.3 Triatomics?

We have recently compared the triple‐bonded 10–VE systems like CO with the 14–VE systems like CPt [38]. In the latter class, there is a well‐defined $\delta^{4}$ ring on Pt, but it has no chance of finding partners on the main‐group C.

We tried to see whether the δ rings could bond to the central U in the uranyl analog ${\mathrm{PtUPt}}^{2+}$, but they did not.

**FIGURE 4:**
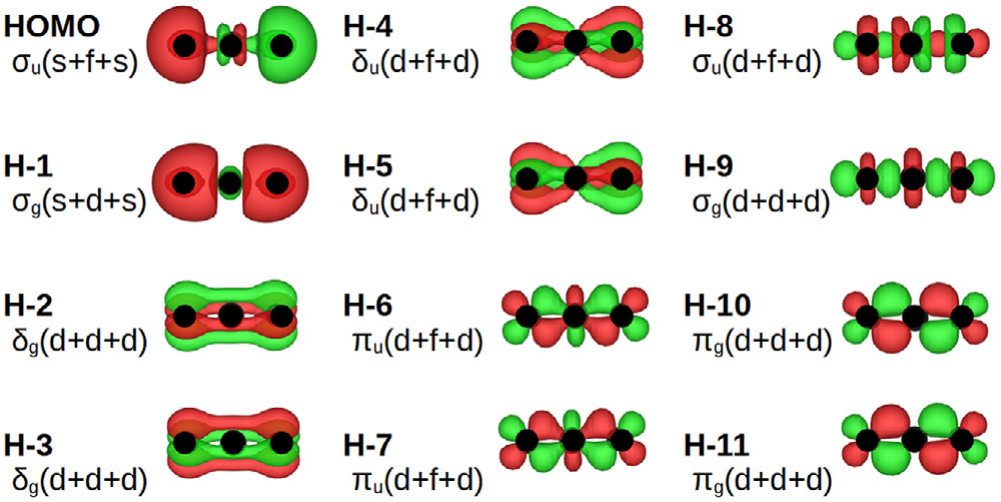
The valence MOs of the 24‐VE species IrUIr.

The NUN analog IrUIr looks better, see Figure 4. With suitable isosurface values, all its 12 valence electrons occupy bonding MOs and thus could contribute to two sixfold bonds. Starting from the top, the highest occupied MO is the Ir 6s‐based $2\sigma_{u}$ and the H‐1 is the $2\sigma_{g}$. The H‐2,3 are the bonding $\delta_{g}$ orbitals, the H‐4,5 the $\delta_{u}$ orbitals, the H‐6,7 the $\pi_{u}$ orbitals and at the bottom the H‐10,11 are the most bonding $1\pi_{g}$ orbitals. The H‐8 is the $1\sigma_{u}$ and the H‐9 the $1\sigma_{g}$ MO, bonded to U 5fσ and 6dσ, respectively. Some of these bonds may be weakish, otherwise this is a school example of hextuple bonding in a triatomic. The calculated R falls between the U‐Ir $\Sigma r_{3}$ and $\Sigma r_{6}$, as seen from Table 5

(3) $${\left(\pi_{g}\right)}^{4}{\left(1\sigma_{g}\right)}^{2}{\left(1\sigma_{u}\right)}^{2}{\left(\pi_{u}\right)}^{4}{\left(\delta_{u}\right)}^{4}{\left(\delta_{g}\right)}^{4}{\left(2\sigma_{g}\right)}^{2}{\left(2\sigma_{u}\right)}^{2},X^{1}\Sigma .$$

**TABLE 5:** Calculated bond lengths, R/pm, and their comparison with sums of covalent radii.

Species	Bond	R	$\Sigma r_{6}$	$\Sigma r_{3}$
IrUIr	Ir‐U	214^a^	209	225
NUIr	Ir‐U	216^a^	209	225
		218.4^b^		

^a^ ORCA BHandHLYP.

^b^ CASPT2 [39].

**FIGURE 5:**
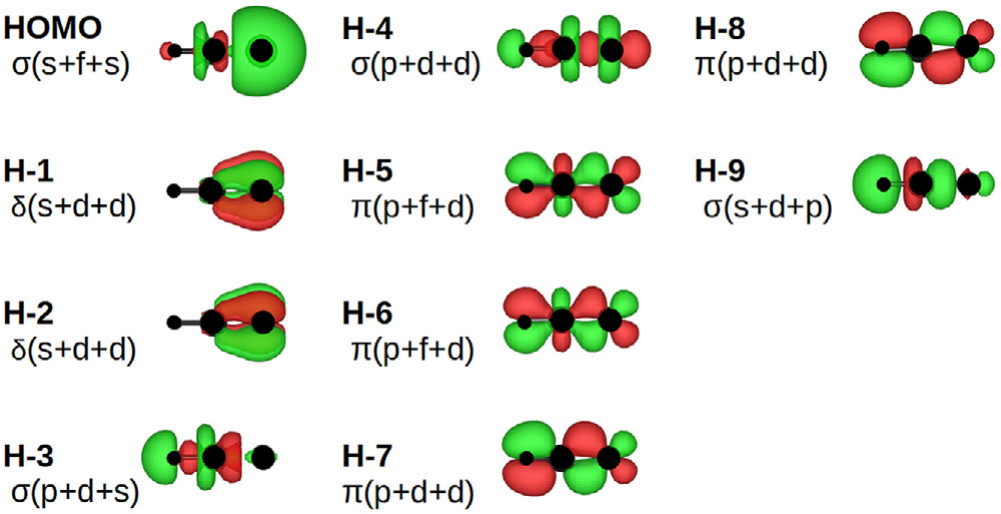
The valence MOs of the 20‐VE species NUIr.

The previously discussed [39] 20‐VE NUIr shows an R(UIr), which also lies between the $r_{6}$ and $r_{3}$ predictions. For its MOs, see Figure 5. Multiple, σ+π+δ U‐Mn bonds in organometallics were discussed in [40]. The Mayer bond order at DFT level was 3.6. Both diatomic CrU and an organometallic example are displayed in their Figure 2.

The calculated R are shown in Table 5.

## 5 Conclusion

We have used accurate experimental data, accurate ab initio data and more approximate DFT data to fit additive covalent radii, $r_{6}$, for over 20 elements from Sc to Pu. When a comparison is possible with Morse's radii, the agreement is good.

## Author Contributions

P. P. initiated the project, wrote the fitting program and performed the Gaussian calculations. MA performed the ORCA calculations. Both authors contributed to all aspects of the manuscript.

## Conflicts of Interest

The authors declare no conflict of interest.

## References

[1] P. Pyykkö and M. Atsumi , “Molecular Single‐Bond Covalent Radii for Elements 1–118,” Chemistry–A European Journal 15 (2009): 186–197. 19058281 10.1002/chem.200800987

[2] P. Pyykkö and M. Atsumi , “Molecular Double‐Bond Covalent Radii for Elements Li–E112,” Chemistry–A European Journal 15 (2009): 12770–12779. 19856342 10.1002/chem.200901472

[3] P. Pyykkö , S. Riedel , and M. Patzschke , “Triple‐Bond Covalent Radii,” Chemistry–A European Journal 11 (2005): 3511–3520. 15832398 10.1002/chem.200401299

[4] P. Pyykkö , “Refitted Tetrahedral Covalent Radii for Solids,” Physical Review B 85 (2012): 024115.

[5] P. Pyykkö , “Additive Covalent Radii for Single‐, Double‐, and Triple‐Bonded Molecules and Tetrahedrally Bonded Crystals: A Summary,” Journal of Physical Chemistry A 119 (2015): 2326–2337. 25162610 10.1021/jp5065819

[6] B. O. Roos , A. C. Borin , and L. Gagliardi , “Reaching the Maximum Multiplicity of the Covalent Chemical Bond,” Angewandte Chemie International Edition 46 (2007): 1469–1472. 17225237 10.1002/anie.200603600

[7] F. A. Cotton and C. B. Harris , “The Crystal and Molecular Structure of Dipotassium Octachlorodirhenate(III) Dihydrate, K_2_[Re_2_C_18_]2H_2_O,” Inorganic Chemistry 4 (1965): 330–333.

[8] F. A. Cotton , C. A. Murillo , and R. A. Walton , Multiple Bonds between Metal Atoms, 3rd ed. (Springer, 2005), 818, See Ch. 8 by R.A. Walton. for Re compounds.

[9] P. Zhang , W.‐L. Zhou , P. Zhang , and S.‐X. Hu , “Electronic Structures and Properties of Actinide‐Bimetal Compounds An_2_O_2_ (An=Th to Cf) and U_2_E_2_ (E=N, F, S),” European Journal of Inorganic Chemistry (2021): 3926–3937.

[10] A. C. Borin , J. P. Gobbo , and B. O. Roos , “A Theoretical Study of the Binding and Electronic Spectrum of the Mo_2_ Molecule,” Chemical Physics 343 (2008): 210–216.

[11] S.‐X. Hu , E. Lu , and S. Liddle , “Prediction of High Bond‐Order Metal–Metal Multiple‐Bonds in Heterobimetallic 3d–4f/5f Complexes $[\text{TM-MN}\left(\text{o-}\left[{\text{NCH}}_{2}P{\left({\mathrm{CH}}_{3}\right)}_{2}\right]{\left(C_{6}H_{4}\right)}_{3}\right]$ (TM = Cr, Mn, Fe; M = U, Np, Pu, and Nd),” Dalton Transactions 48 (2019): 12867–12879. 31389454 10.1039/c9dt03086g

[12] O. Krechkivska and M. D. Morse , “ZrFe, a Sextuply‐Bonded Diatomic Transition Metal?,” Journal of Physical Chemistry A 117 (2013): 992–1000. 22452673 10.1021/jp301096z

[13] M. D. Morse , “Clusters of Transition‐Metal Atoms,” Chemical Reviews 86 (1986): 1049–1109.

[14] Y. M. Efremov , A. N. Samoilova , and L. V. Gurvich , “The λ = 4600 Å band in a Spectrum by Pulsed Photolysis of Chromium Carbonyl,” Optics and Spectroscopy 36 (1974): 381–382, Russian original 36, 654‐657.

[15] V. E. Bondybey and J. E. English , “Electronic Structure and Vibrational Frequency of Cr_2_ ,” Chemical Physics Letters 94 (1983): 443–447.

[16] M. Brynda , L. Gagliardi , and B. O. Roos , “Analysing the Chromium–Chromium Multiple Bonds Using Multiconfigurational Quantum Chemistry,” Chemical Physics Letters 471 (2009): 1–10.

[17] Y.‐L. Wang , H.‐S. Hu , W.‐L. Li , F. Wei , and J. Li , “Relativistic Effects Break Periodicity in Group 6 Diatomic Molecules,” Journal of the American Chemical Society 138 (2016): 1126–1129. 26787134 10.1021/jacs.5b11793

[18] F. Ruipérez , G. Merino , J. M. Ugalde , and I. Infante , “Molecules With High Bond Orders and Ultrashort Bond Lengths: CrU, MoU, and WU,” Inorganic Chemistry 52 (2013): 2838–2843. 23464540 10.1021/ic301657c

[19] M. J. Frisch , G. W. Trucks , H. B. Schlegel , et al., Gaussian 16 Revision C.01 (Gaussian Inc. Wallingford CT, 2016).

[20] F. Neese , F. Wennmohs , U. Becker , and C. Ripplinger , “The ORCA Quantum Chemistry Program Package,” Journal of Chemical Physics 152 (2020): 224108. 32534543 10.1063/5.0004608

[21] F. Ruipérez , J. M. Ugalde , and I. Infante , “Electronic Structure and Bonding in Heteronuclear Dimers of V, Cr, Mo, and W: A CASSCF/CASPT2 Study,” Inorganic Chemistry 50 (2011): 9219–9229. 21894920 10.1021/ic200061h

[22] O. Krechkivska , M. D. Morse , A. Kalemos , and A. Mavridis , “Electronic Spectroscopy and Electronic Structure of Diatomic TiFe,” Journal of Chemical Physics 137 (2012): 054302. 22894343 10.1063/1.4738958

[23] Y. M. Efremov , A. N. Samoilova , V. B. Kozhukovsky , and L. V. Gurvich , “On the Electronic Spectrum of the Mo_2_ Molecule Observed After Flash Photolysis of Mo(CO),” Journal of Molecular Spectroscopy 73 (1978): 430–440.

[24] J. B. Hopkins , P. R. R. Langridge‐Smith , M. D. Morse , and R. E. Smalley , “Supersonic Metal Cluster Beams of Refractory Metals: Spectral Investigations of Ultracold Mo_2_ ,” Journal of Chemical Physics 78 (1983): 1627–1637.

[25] A. C. Borin , J. P. Gobbo , and B. O. Roos , “Electronic Structure and Chemical Bonding in W_2_ Molecule,” Chemical Physics Letters 490 (2010): 24–28.

[26] E. M. Spain , J. M. Behm , and M. D. Morse , “The A ^1^Σ^+^ ← X ^1^Σ^+^ band system of CrMo,” Chemical Physics Letters 179 (1991): 411–416.

[27] D. J. Matthew , S. H. Oh , A. Sevy , and M. D. Morse , “The Bond Length and Bond Energy of Gaseous CrW,” Journal of Chemical Physics 144 (2016): 214306. 27276956 10.1063/1.4952453

[28] G. L. Gutsev , M. D. Mochena , P. Jena , C. W. Bauschlicher Jr. , and H. Partridge III , “Periodic Table of 3d‐Metal Dimers and Their Ions,” Journal of Chemical Physics 121 (2004): 6785–6797. 15473736 10.1063/1.1788656

[29] M. A. Baudhuin , P. Boopalachandran , S. Rajan , and D. G. Leopold , “A Study of NbMo and NbMo‐ by Anion Photoelectron Spectroscopy,” Journal of Physical Chemistry A 125 (2021): 9658–9679. 34723518 10.1021/acs.jpca.1c07669

[30] P. Pyykkö , “Magically Magnetic Gadolinium,” Nature Chemistry 7 (2015): 680–680, an html version is available at, http://www.nature.com/nchem/journal/v7/n8/full/nchem.2287.html. Further historical details are given in a blog entry:, http://blogs.nature.com/thescepticalchymist/2015/07/more‐on‐gadolinium.html. 10.1038/nchem.2287 26201746

[31] R. Nagarajan and M. D. Morse , “ $\Pi_{1}\leftarrow X\Sigma^{+}\;1$ Band Systems of Jet‐Cooled ScCo and YCo,” Journal of Chemical Physics 127 (2007): 074304. 17718610 10.1063/1.2756533

[32] R. Nagarajan , S. M. Sickafoose , and M. D. Morse , “Rotationally Resolved Spectra of Jet‐Cooled VMo,” Journal of Chemical Physics 127 (2007): 014311. 17627350 10.1063/1.2747617

[33] L. Gagliardi , P. Pyykkö , and B. O. Roos , “A Very Short Uranium–Uranium Bond: The Predicted Metastable ${U_{2}}^{2+}$ ,” Physical Chemistry Chemical Physics 7 (2005): 2415–2417. 15962023 10.1039/b505593h

[34] H. Dong , Q.‐Y. Meng , B.‐Z. Chen , and Y.‐B. Wu , “Theoretical Studies on the Multiple Metal–Metal Bonds in the Bimetallic Molecules and the Ultrashort V‐Mn Bonds in the Complexes,” Journal of Organometallic Chemistry 717 (2012): 108–115.

[35] D. Sundholm , P. Pyykkö , and L. Laaksonen , “Fully numerical HFS calculations on Cr_2_: Basis‐set truncation error on the bond length and interaction of the semicore orbitals,” Finnish Chemical Letters 1985 (1985): 51–55.

[36] H. Mahdavi , V. Honavar , and D. Morgan , “Beyond Training Data: How Elemental Features Enhance ML‐Based Formation Energy Predictions,” Digital Discovery 4 (2025): 2972–2982.

[37] F. Ruiperez , M. Piris , J. M. Ugalde , and J. M. Matxain , “The Natural Orbital Functional Theory of the Bonding in Cr_2_, Mo_2_ and W_2_ ,” Physical Chemistry Chemical Physics 15 (2013): 2055–2062. 23262452 10.1039/c2cp43559d

[38] M. Atsumi and P. Pyykkö , “10‐Valence‐Electron C≡O and the 14‐VE C≡Pt: Two Triple‐Bonded Isoelectronic Families Differing by a $d\delta^{4}$ Ring,” Inorganic Chemistry 62 (2023): 21083–21090, 10.1021/acs.inorgchem.3c02889. 38050990 PMC10751794

[39] L. Gagliardi and P. Pyykkö , “Theoretical Search for Very Short Metal‐Actinide Bonds: NUIr and Isoelectronic Systems,” Angewandte Chemie International Edition 43 (2004): 1573–1576. 10.1002/anie.200353261 15022237

[40] P. Sharma , D. R. Pahls , B. I. Ramirez , C. C. Lu , and L. Gagliardi , “Multiple Bonds in Uranium–Transition Metal Complexes,” Inorganic Chemistry 58 (2019): 10139–10147. 31329432 10.1021/acs.inorgchem.9b01264

